# Talaporfin Sodium as a Clinically Translatable Radiosensitizer in Radiodynamic Therapy

**DOI:** 10.3390/biom15121748

**Published:** 2025-12-18

**Authors:** Junko Takahashi, Junkoh Yamamoto, Kohei Suzuki, Shohei Nagasaka, Kaizhen Yang, Haobo Zhao, Teppei Yamaoka

**Affiliations:** 1 Graduate School of Information, Production and Systems, Waseda University, Kitakyusyu, Fukuoka 808-0135, Japan; 2 Department of Neurosurgery, University of Occupational and Environmental Health, Kitakyushu, Fukuoka, 807-8555, Japan

**Keywords:** radiodynamic therapy (RDT), radiotherapy, photodynamic therapy (PDT), talaporfin sodium, mono-L-aspartyl chlorin e6 (NPe6), pancreatic cancer, RNA-seq

## Abstract

Talaporfin sodium (mono-L-aspartyl chlorin e6; NPe6), a second-generation photosensitizer, is clinically used in photodynamic therapy (PDT). It accumulates preferentially in tumors and exhibits deep tissue penetration, rapid systemic clearance, and minimal photosensitivity. However, treatment of deep-seated malignancies remains challenging. Here, we demonstrate that talaporfin sodium undergoes physicochemical reactions with X-rays to generate reactive oxygen species, a mechanism analogous to that of 5-aminolevulinic acid (5-ALA)-induced protoporphyrin IX in radiodynamic therapy (RDT). To evaluate its therapeutic efficacy, we employed a pancreatic cancer xenograft model using MIA PaCa-2 cells in mice. Talaporfin sodium was administered intravenously 2 h before X-ray exposure, followed by fractionated X-ray irradiation (3 Gy daily for 3 consecutive days). Talaporfin-mediated RDT significantly inhibited tumor growth compared with radiation therapy alone. Furthermore, an exploratory RNA-seq analysis of xenografts revealed transcriptional signatures of stress and immune activation, suggesting that talaporfin-mediated RDT enhances oxidative and immunogenic responses within the tumor microenvironment. These findings highlight the potential of talaporfin sodium as a clinically translatable radiosensitizer for RDT, offering a promising strategy for the treatment of deep-seated cancers such as pancreatic carcinoma.

## 1. Introduction

Radiotherapy (RT) is one of the standard modalities for the treatment of various malignant tumors, along with surgery and chemotherapy. It provides effective local tumor control and is minimally invasive. However, several challenges remain in achieving optimal therapeutic outcomes. A major limitation is that adequate radiation doses cannot always be delivered to tumors located adjacent to critical normal tissues or organs at risk, owing to the need to prevent radiation-induced injury to healthy structures [1]. Furthermore, highly infiltrative or radioresistant tumors often respond poorly to RT alone, resulting in insufficient tumor control and frequent local recurrence [2,3]. These limitations underscore the need for novel strategies to enhance the therapeutic efficacy of RT without increasing toxicity to surrounding normal tissues.

To overcome these issues, radiosensitization therapies have been investigated as a means to enhance the biological effects of radiation. Among them, radiodynamic therapy (RDT), which combines ionizing radiation with photosensitizing agents traditionally used in photodynamic therapy (PDT), has recently attracted attention [4,5,6,7]. In RDT, radiosensitizers are activated by radiation instead of light, generating reactive oxygen species (ROS) that induce cytotoxicity in tumor cells. This mechanism is of particular interest because it may be effective even under hypoxic conditions, which are known to reduce radiation efficacy [8].

Currently, 5-aminolevulinic acid (5-ALA) is one of the most widely used photosensitizers in clinical practice, particularly for photodynamic diagnosis (PDD) of malignant gliomas and other brain tumors. Owing to the well-established knowledge of its preferential accumulation in malignant gliomas, 5-ALA-based RDT has been the focus of active preclinical and clinical investigations [9,10].

While numerous compounds have been screened for their potential as agents in RDT, a comprehensive understanding of the physicochemical and radiochemical processes responsible for radiosensitization is still lacking [11]. Furthermore, it is not yet established whether all photosensitizers currently used in PDT can be effectively translated to RDT, since their interactions with ionizing radiation may differ in terms of excitation mechanisms, reactive species generation, and radiochemical stability.

Talaporfin sodium (mono-L-aspartyl chlorin e6; NPe6, hereafter referred to as talaporfin), a second-generation photosensitizer, exhibits strong photodynamic efficacy with a high singlet oxygen quantum yield [12,13], together with favorable tumor selectivity and rapid clearance from normal tissues [14,15]. In Japan, talaporfin (commercially available as Laserphyrin*^®^*) has been clinically approved for PDT in patients with centrally located early-stage squamous-cell lung carcinoma and for the treatment of primary malignant brain tumors [16,17,18]. Given its favorable photochemical characteristics, tumor selectivity, and established clinical use, talaporfin represents a promising candidate as a radiosensitizer for RDT. In this study, we investigated the therapeutic efficacy and molecular mechanisms of talaporfin-mediated RDT, focusing on its potential to enhance radiation-induced oxidative stress and antitumor immune responses. Our findings provide new insight into the development of clinically translatable radiosensitization strategies for deep-seated or radioresistant malignancies.

## 2. Materials and Methods

### 2.1. Materials

Talaporfin (generic name: talaporfin sodium; trade name: Laserphyrin; chemical name: mono-L-aspartyl chlorin e6 (NPe6), (+)-tetrasodium (2S,3S)-18-carboxylato-20-[N-(S)-1,2-dicarboxylatoethyl]carbamoylmethyl-13-ethyl-3,7,12,17-tetramethyl-8-vinylchlorin-2-propanoate; molecular formula: C_38_H_37_N_5_Na_4_O_9_; molecular weight: 799.69) was provided by Meiji Seika Pharma Co., Ltd. (Tokyo, Japan). 3′-(p-Aminophenyl) fluorescein (APF) was purchased from Goryo Chemical (Sapporo, Japan). Dihydroethidium (DHE), methanol, Dulbecco’s modified Eagle’s medium (DMEM), PBS buffer, penicillin, streptomycin and crystal violet were purchased from Fujifilm Wako Industries Ltd. (Osaka, Japan). Lyso-Tracker Green, Mito-Tracker Green, and Hoechst^®^ 33342 were purchased from Thermo Fisher Scientific (Waltham, MA, USA).

### 2.2. X-Ray Irradiation

X-ray irradiation was performed using an MBR-1520R-4 irradiator (Hitachi Power Solutions, Hitachi, Japan) at 150 kV and 20 mA with added filtration of 0.5 mm Al and 0.1 mm Cu. The dose rate was 1.0 Gy/min at the sample stage.

### 2.3. Evaluation of ROS Production

To assess the formation of hydroxyl radicals (^•^OH), APF was employed as the fluorescent probe [19]. Fluorescence was recorded using a Synergy H1 plate reader (Agilent Technologies, Santa Clara, CA, USA) with excitation at 490 nm and emission at 515 nm. Reaction mixtures were prepared in black 96-well plates to a final volume of 100 µL, containing talaporfin at 0, 0.03, 0.1, or 0.3 µM, APF at 5 µM, and PBS buffer. All experimental steps involving talaporfin were conducted under dark conditions to eliminate any unintended photodynamic activation. The prepared reaction mixtures were then irradiated at 0, 3, 5, or 10 Gy followed by fluorescence measurement. Superoxide radicals (O2^•−^) were detected using DHE as the fluorescent probe [20]. Fluorescence was measured with excitation at 485 nm and emission at 610 nm. Reaction mixtures were prepared in the same manner as for ^•^OH detection, except that APF (5 µM) was replaced with DHE at 50 µM.

### 2.4. Cell Culture

MIA PaCa-2 human pancreatic cancer cells and U-251 MG glioblastoma cells (U-251 MG-Luc) were obtained from the Japanese Collection of Research Bioresources (Osaka, Japan). The cell lines were cultured in DMEM supplemented with 10% FBS in a 5% CO_2_ humidified incubator at 37 °C. The medium was supplemented with 100 units/mL of penicillin and 100 μg/mL of streptomycin.

### 2.5. Clonogenic Assay

Cells were incubated in complete medium supplemented with 0, 3, 10, or 30 μg/mL talaporfin for 4 h under dark conditions, followed by washing with PBS. Afterward, the medium was replaced with fresh culture medium, and the cells were irradiated at a rate of 1 Gy/min. Following exposure to 0, 2, 4, or 6 Gy of X-ray irradiation, the cells were transferred into 25-cm^2^ flasks at a density of 1000 cells per flask and maintained at 37 °C in a 5% CO_2_ atmosphere. Cell survival was assessed using a clonogenic assay. Colonies were fixed and stained with 2% crystal violet in methanol after a minimum of 14 days of incubation. Only colonies consisting of more than 50 cells were counted as viable.

### 2.6. Imaging of the Intracellular Localization of Talaporfin

The subcellular localization of Talaporfin in conjunction with the fluorescence signals from organelle-specific probes, was visualized. Cells were seeded in glass-bottom culture dishes (35 mm in diameter) at a density of 2 × 10^5^ cells/well density. Subsequently, Talaporfin was added to the culture medium to a final concentration of 30 μg/mL and incubated for 3.5 h in the dark. Cells were then stained for 30 min with LysoTracker Green (50 nM), MitoTracker Green (100 nM), and Hoechst 33342 (1 μg/mL), respectively. After staining, the cells were washed twice with PBS. Images were acquired using a laser scanning confocal microscope FV3000 (Olympus Corporation, Tokyo, Japan) equipped with a 100× oil-immersion objective. Talaporfin fluorescence was excited at 405 nm and emission was collected between 650 and 750 nm. Mitochondria and lysosomes were visualized using organelle-specific fluorescent dyes excited at 488 nm with emission collection between 500 and 600 nm, while the nuclear stain was excited at 405 nm with emission collected between 450 and 550 nm. No ROS-sensitive probes were used in these imaging experiments.

### 2.7. In Vivo Evaluation of Talaporfin-Mediated RDT in Tumor-Bearing MIA PaCa-2 Xenografts

Human pancreatic cancer MIA PaCa-2 were used to generate subcutaneous xenografts. Six-week-old female nBALB/c nu/nu mice (Charles River Laboratories Japan, Inc., Yokohama, Japan) were anesthetized, and 2.0 × 10^6^ MIA PaCa-2 cells were injected subcutaneously. Mice were maintained under a controlled conventional environment at 25 ± 1 °C, 50 ± 10% humidity, and a 12 h light/dark cycle, with free access to standard chow and water. Paper bedding and environmental enrichment were provided. Tumor volume before treatment initiation was kept below approximately 400 mm^3^, in compliance with the institutional humane endpoint of 1000 mm^3^. This pre-treatment volume was chosen because it provides sufficient tumor mass to allow reliable assessment of treatment-induced regression while remaining well within ethical limits. After tumor volume reached approximately 400 mm^3^, mice were divided into four groups to ensure tumor volume uniformity: (1) NT, control group (n = 4); (2) TS, talaporfin (10 mg/kg, i.v.) only (n = 4); (3) XT, X-ray irradiation only (total dose, 9 Gy; 3 Gy once daily for three consecutive days) (n = 7); (4) TS-XT, talaporfin (10 mg/kg, i.v.) administered 2 h before each irradiation (n = 9). For X-ray exposure, each mouse was placed in a plastic restrainer designed with an opening positioned above the tumor region. A collimated X-ray beam was directed to a 20 × 20 mm irradiation field centered on the tumor, ensuring full coverage of the entire tumor mass. Mice in the X-ray treatment groups received talaporfin (10 mg/kg, i.v.) diluted in PBS 2 h before each irradiation. Mice without X-ray irradiation received an equivalent volume of PBS (vehicle control). To exclude any photodynamic effect, all mice—including vehicle controls—were maintained under light-shielded conditions for at least 24 h after intravenous administration. Tumor volume was estimated weekly from caliper measurements using the formula: volume = (shortest diameter)^2^ × (longest diameter) × 0.5. Body weight and tumor dimensions were monitored and recorded at weekly intervals following cell implantation. Mice were sacrificed 25 days after the last X-ray irradiation. Mice in NT and TS groups, which did not receive radiation, were euthanized approximately 17 days after last irradiation because their tumor volumes continued to increase. The tumors were weighed using an electronic balance, immediately immersed in RNAlater (Qiagen, Hilden, Germany), and stored at −80 °C until RNA extraction. The number of animals per group was determined based on preliminary data to ensure statistical robustness and reproducibility while adhering to the 3Rs principle. All animal procedures were reviewed and approved by the Animal Experimentation Committee of the University of Occupational and Environmental Health, Japan (Approval No. AE11-02).

### 2.8. RNA-Seq

#### 2.8.1. RNA-Seq Data Acquisition

Total RNA was extracted from xenograft tumors using the RNeasy Mini Kit (Qiagen) according to the manufacturer’s instructions. RNA integrity was assessed using an Agilent 2100 Bioanalyzer (Agilent Technologies, Santa Clara, CA, USA; RIN > 7.0). Poly(A)-enriched libraries were prepared using the TruSeq Stranded mRNA Library Prep Kit (Illumina, San Diego, CA, USA). Libraries were quantified, pooled, and sequenced on an Illumina NovaSeq 6000 platform (Illumina, San Diego, CA, USA) to generate paired-end 150 bp reads (approximately 6 Gb per sample, ~20 million read pairs).

#### 2.8.2. RNA-seq Data Processing and Differential Expression Analysis

Raw sequencing reads were subjected to quality control using FastQC v0.11.9 (Babraham Bioinformatics, Cambridge, UK), followed by adapter and quality trimming with Trim Galore v0.6.10 (Babraham Bioinformatics, Cambridge, UK). Transcript quantification was performed using Salmon v1.10.1 (COMBINE-lab, University of Maryland, College Park, MD, USA) in quasi-mapping mode against both the human (GENCODE release 42, GRCh38.p13) and mouse (GENCODE release M35, GRCm39) transcriptome references. TPM (Transcripts Per Million) and NumReads values were obtained from the Salmon output (quant.sf files). Transcript-level abundances were summarized to gene-level expression values using tximport v1.30.0 (Bioconductor project) in R v4.3.2 (R Foundation for Statistical Computing, Vienna, Austria). All analyses were performed using default parameters unless otherwise specified. The false discovery rate (FDR) was calculated using the limma package v3.58.1 (Bioconductor project). Genes with unadjusted *p* < 0.01 (Student’s *t*-test) were selected for exploratory functional analyses, including Gene Ontology (GO) enrichment and pathway grouping. The proportion of mouse-derived reads was defined as the mouse read fraction, which represents the degree of host RNA admixture in each xenograft sample. Gene symbols follow the official nomenclature for each species: HGNC for human genes and MGI for mouse genes.

#### 2.8.3. Data Availability

RNA-seq data have been deposited in the Gene Expression Omnibus (GEO) under the accession number GSE312155.

### 2.9. Statistics

Group-level statistical differences were evaluated using a one-way ANOVA. The assumption of equal variances was examined with Bartlett’s test. If homogeneity of variance was confirmed (*p* ≥ 0.05), post hoc analyses were conducted using Tukey’s honestly significant difference test. In cases where variances were not uniform (*p* < 0.05), the Games–Howell test was employed. Statistical significance was defined as *p* < 0.05. Relationships between gene expression profiles were quantified using Pearson correlation coefficients.

## 3. Results

### 3.1. Talaporfin Enhances ROS Generation upon X-Ray Irradiation

To confirm that the interaction between talaporfin and X-rays generates ROS, ROS production was evaluated using two fluorescence probes, APF and DHE, which primarily detect hydroxyl radicals (^•^OH) and superoxide anions (O_2_^•−^), respectively, although they may also respond to other ROS.

**Figure 1:**
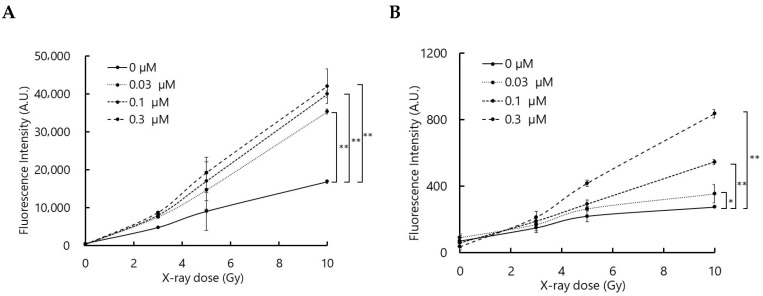
Talaporfin enhances ROS generation upon X-ray irradiation. ROS production was assessed using (**A**) APF for ^•^OH and (**B**) DHE for O_2_^•−^. Fluorescence intensity increased with X-ray dose in the presence of talaporfin, although the relationship with talaporfin concentration was not strictly proportional. (Data are presented as mean ± SD, n = 4; * *p* < 0.05, ** *p* < 0.01).

As shown in Figure 1, the fluorescence intensities of APF and DHE increased with X-ray dose in the presence of talaporfin. Although the relationship between fluorescence intensity and talaporfin concentration was not strictly proportional, the overall trend suggests that talaporfin facilitates ROS generation upon X-ray irradiation.

### 3.2. Talaporfin Enhanced Cellular Responses to X-Ray Irradiation

**Figure 2:**
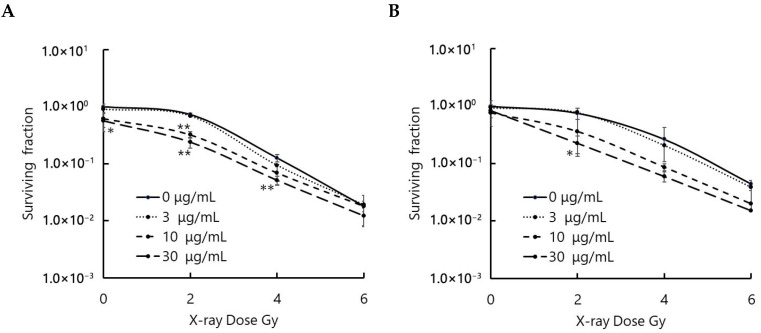
Radiation response of (**A**) MIA PaCa-2 and (**B**) U-251 MG cells following pretreatment with talaporfin. Cells were incubated with 0, 3, 10, or 30 µg/mL talaporfin for 4 h in light-protected conditions, after which the compound-containing medium was replaced with drug-free medium. X-ray irradiation was then applied at 1 Gy/min to achieve the indicated doses. Surviving fractions were quantified by clonogenic assays and expressed as mean ± SD (n = 4). Statistical comparisons at each radiation dose were performed relative to the corresponding control group (* *p* < 0.05, ** *p* < 0.01).

The combined effects of talaporfin and ionizing radiation on the human pancreatic cell line MIA PaCa-2 and the human glioblastoma cell line U-251 MG were examined in vitro using a clonogenic assay (Figure 2). Both cell lines were incubated with talaporfin for 4 h prior to irradiation. Error bars on the survival curves indicate the standard deviation derived from four independent experiments. The results showed that X-ray irradiation suppressed the growth of both cell lines in a dose-dependent manner, and talaporfin further enhanced this inhibitory effect. To quantitatively evaluate this radiosensitizing effect, dose enhancement factors at 10% survival (DEF_0_._1_) were calculated from the dose–response curves, where DEF is defined as the ratio of doses producing the same survival fraction in the absence and presence of talaporfin. In MIA PaCa-2 cells, the DEF_0_._1_ values were 1.13, 1.20, and 1.29 at 3, 10, and 30 µg/mL talaporfin, respectively. In U-251 MG cells, the corresponding DEF_0_._1_ values were 1.04, 1.41, and 1.57. These findings suggest that talaporfin potentiates radiation-induced cytotoxicity in both cell lines.

### 3.3. Intracellular Localization of Talaporphyrin In Vitro

**Figure 3:**
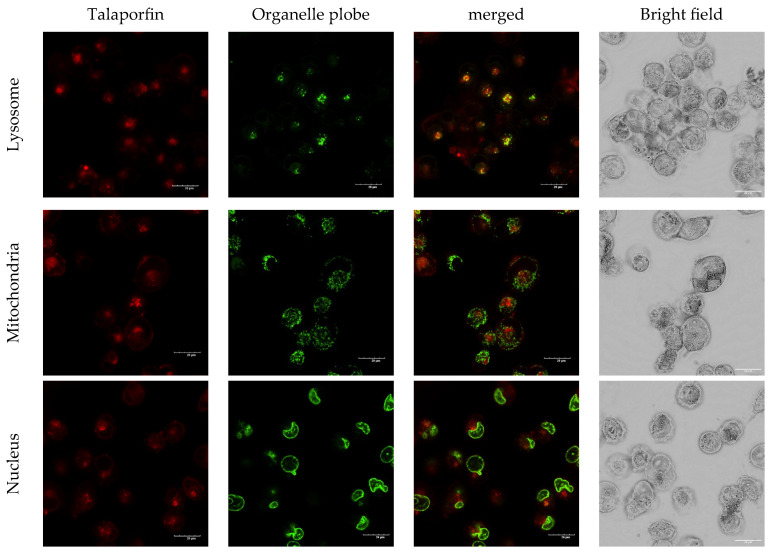
Cells were incubated with talaporfin (30 μg/mL) for 3.5 h in the dark, followed by staining with LysoTracker Green (50 nM), MitoTracker Green (100 nM), and Hoechst 33342 (1 μg/mL) for 30 min. After washing twice with PBS, fluorescence images were acquired using a laser scanning confocal microscope (FV3000; Olympus, Tokyo, Japan) equipped with a 100× oil-immersion objective. Talaporfin fluorescence (red) largely colocalized with the lysosomal probe (green; yellow in merged images), whereas minimal overlap with mitochondrial or nuclear probes was observed. Bright-field images were digitally processed to enhance cellular morphological visibility. They are provided to illustrate overall morphology rather than detailed subcellular localization. Scale bar = 20 μm.

To determine the intracellular localization of talaporfin, cells were co-stained with probes for lysosomes, mitochondria, and nuclei. In the images stained with organelle probes, talaporfin fluorescence appeared in red, whereas the lysosomal, mitochondrial, and nuclear probes were visualized in green; overlapping signals appeared yellow in the merged images, indicating that talaporfin was predominantly localized within lysosomes. However, the overlap was not complete, suggesting that a fraction of talaporfin was present outside lysosomes. In contrast, little to no colocalization was observed with mitochondrial or nuclear markers (Figure 3).

### 3.4. Talaporfin Enhanced Tumor Response to X-Ray Irradiation In Vivo

To evaluate the therapeutic efficacy of talaporfin-mediated RDT, a tumor-bearing mouse model was established by subcutaneous implantation of MIA PaCa-2 (pancreatic cancer) cells into BALB/c nu/nu mice. When the tumor volume reached approximately 400 mm^3^, talaporfin (10 mg/kg) was intravenously administered via the tail vein two hours before each irradiation. Mice were irradiated with 3 Gy per day for three consecutive days, for a total dose of 9 Gy.

**Figure 4:**
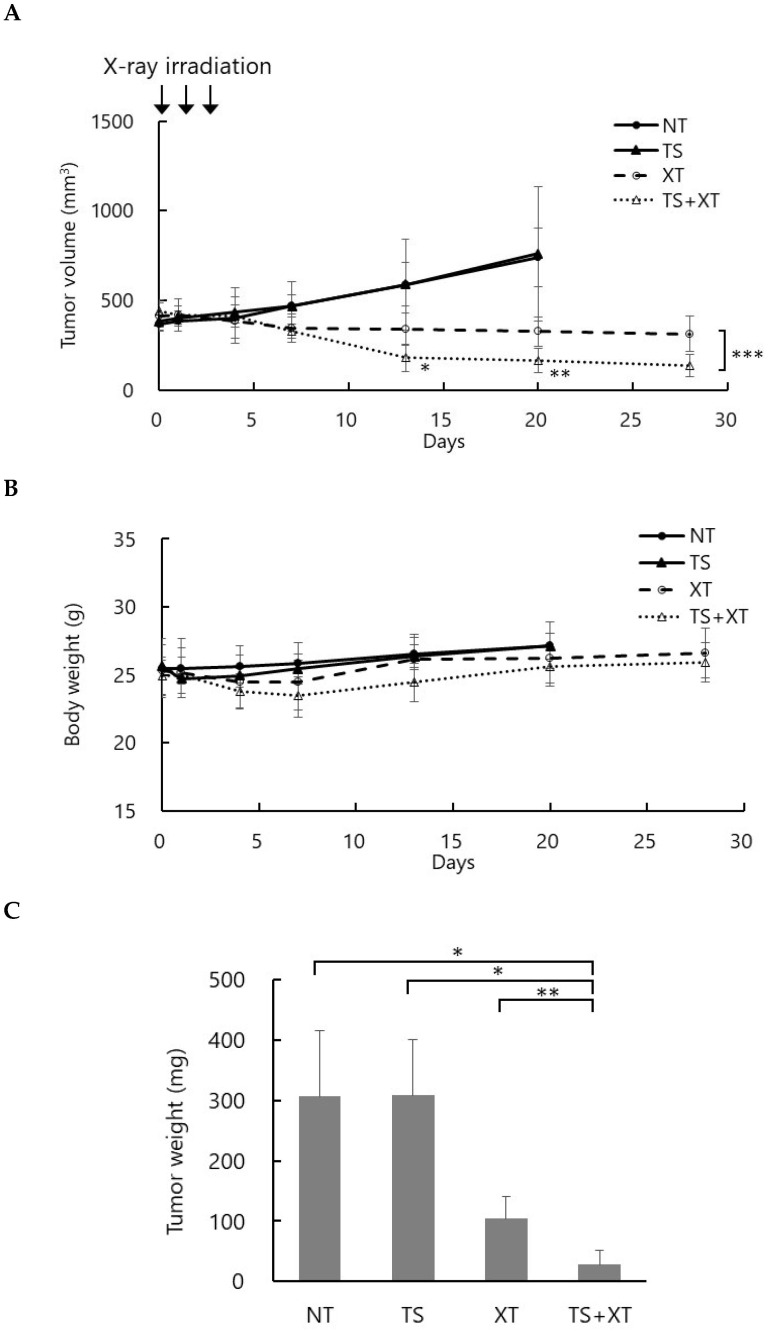
In vivo antitumor effects of talaporfin combined with fractionated X-ray irradiation. (**A**) Tumor growth curves of MIA PaCa-2 xenografts under different treatment conditions: no treatment (NT), talaporfin alone (TS; 10 mg/kg), X-ray alone (XT; 9 Gy total; 3 Gy per day for three consecutive days), and talaporfin 10 mg/kg + X-ray (TS-XT). Tumor volumes were monitored for 25 days after the last X-ray irradiation. In the XT group, tumor growth was moderately suppressed, whereas in the TS-XT group, tumor growth was markedly inhibited, with some tumors showing regression. In the NT and TS groups, animals were euthanized 17 days after the last radiation dose due to continuous tumor growth. (**B**) Body weight changes during the experimental period. No significant body weight loss was observed in any treatment group. (**C**) Final tumor weights of MIA PaCa-2 xenografts at the end of the experiment. Tumor weights were significantly lower in the TS-XT group compared with the XT group. Data are presented as mean ± SD (n = 4–9). * *p* < 0.05, ** *p* < 0.01, *** *p* < 0.001 versus the corresponding XT group in (**A**); * *p* < 0.05, ** *p* < 0.01 among groups in (**C**).

Figure 4 shows changes in tumor volume over time (A), body weight (B), and tumor weight at necropsy (C). The observation period was 25 days after the last irradiation. In the NT (no treatment) and TS (talaporfin alone) groups, tumor volumes continuously increased, and mice were euthanized 17 days after the last irradiation upon reaching the humane endpoint (~1000 mm^3^). In the XT (X-rays alone) group, tumor growth was moderately suppressed, whereas in the TS-XT (talaporfin + X-rays) group, tumor growth was markedly inhibited, with some tumors showing regression (Figure 4A). Both the XT and TS-XT groups exhibited a transient decrease in body weight following irradiation; however, body weight subsequently recovered, and no significant difference was observed among the groups (Figure 4B). At the end of the experiment, the mean tumor weights were as follows: NT, 305.9 ± 95.4 mg; TS, 307.9 ± 79.7 mg; XT, 103.9 ± 33.8 mg; and TS-XT, 28.4 ± 21.7 mg. These results demonstrate that combined treatment with talaporfin and X-ray irradiation significantly enhanced tumor growth suppression compared with either treatment alone, indicating a radiosensitizing effect of talaporfin under X-ray exposure (Figure 4C).

### 3.5. Talaporfin-Mediated RDT Induced Transcriptomic Changes in Tumors

Comprehensive gene expression profiling was performed on tumor tissues collected 25 days after the last X-ray irradiation. RNA-seq analyses were conducted for NT, TS, XT, and TS + XT groups. Three biological replicates per group were selected for RNA-seq analysis, ensuring representative tumor morphology and volume within each group. Quality control of RNA-seq data revealed that most samples contained 26–40% mouse-derived reads, consistent with the expected level of stromal contribution in xenografts (Table 1). One TS + XT sample exhibited a markedly higher mouse read fraction (77.5%), likely reflecting host tissue admixture associated with extensive tumor regression.

Pearson’s correlation coefficients were calculated from normalized expression values across all 12 RNA-seq datasets for both human- and mouse-derived reads. A color-coded pairwise correlation matrix for each species is shown in Supplementary Figure S1, where the color intensity indicates the strength of the correlation (red, high; blue, low). The correlation heatmaps demonstrated strong intra-group reproducibility among biological replicates and a progressive decrease in inter-group correlations from NT and TS to XT and TS + XT. This trend indicated distinct transcriptional reprogramming induced by radiation and the radiosensitizer. One TS + XT sample exhibited a relatively lower correlation with the other replicates, consistent with its high mouse RNA admixture due to extensive tumor regression. These results confirmed the reliability and biological consistency of the RNA-seq dataset, supporting subsequent differential expression and functional enrichment analyses.

Although differential expression analysis was initially performed using false discovery rate (FDR)-adjusted criteria, only a limited number of genes were identified as significantly altered between groups. This restricted detection was likely attributable to biological heterogeneity, including the influence of mouse RNA admixture in the TS + XT group. To further interpret transcriptomic alterations associated with TS + XT-induced tumor regression, genes showing nominal *p*  <  0.01 differences between NT and TS + XT were categorized by biological function for both human- and mouse-derived reads. The categorized gene lists are summarized in Table 2, and the complete gene lists for each category are provided in Supplementary Tables S1–S4.

In human (tumor) cells, upregulated genes were mainly associated with proteostasis, DNA repair, and immune activation. Increased expression of *PSMD13*, *PSMC4*, and *AKR1A1* indicates enhanced protein quality control and oxidative stress defense. The replication stress gene *PRIMPOL* and inflammatory mediators *CXCL1*, *CXCL2*, and *MICA* were also induced, reflecting activation of DNA repair and immune pathways. Conversely, genes involved in mitochondrial metabolism and developmental transcription were suppressed. Downregulation of *DMGDH*, *ATP23*, and *COQ3* suggests reduced oxidative phosphorylation, while loss of *TBR1*, *HOXD12*, and *SATB1* points to repression of stemness- and differentiation-related programs. Cytoskeletal components such as *MTCL1* and *DNAH1* were also decreased, implying attenuated cellular motility.

In mouse tissues, the integrated stress response was prominently activated. Upregulation of *Gstp1*, *Slc39a3*, and *Ddit3* denotes engagement of oxidative and ER stress pathways, accompanied by metabolic reprogramming marked by *Rbks* and *Enho*. In contrast, angiogenic and DNA repair regulators—including *Spon2*, *Sema3a*, *Myh10*, *Setd1a*, and *Msh6*—were downregulated, along with mitochondrial energy genes such as *Ndufs1* and *Vma21*.

These transcriptional changes collectively suggest that the host microenvironment underwent suppression of angiogenic and structural pathways, concurrent with activation of stress response signaling. Taken together, both human and mouse transcriptomes exhibited coordinated expression patterns consistent with immunogenic stress induction and stromal reprogramming following combined TS + XT treatment, supporting the observed tumor regression at the phenotypic level.

## 4. Discussion

Talaporfin is a clinically approved second-generation photosensitizer for PDT in Japan, characterized by its strong singlet oxygen generation and rapid clearance from normal tissues [12,13,14,15,16,17,18]. Although PDT has proven effective in various cancers, its therapeutic application remains constrained by the limited penetration depth of excitation light. This intrinsic limitation has inspired the development of RDT, a conceptually analogous modality in which radiosensitizers are activated by ionizing radiation rather than visible light [4,5,6,7]. Because RDT is driven by radiation rather than photonic excitation, it offers a potential strategy to treat deep-seated malignancies that are otherwise inaccessible to PDT. However, unlike red light, ionizing radiation is accompanied by well-recognized risks, including normal tissue damage and carcinogenic potential, which must be carefully considered when evaluating the clinical applicability of RDT. Recent studies have shown that several photosensitizers can function as efficient radiosensitizers through ROS generation upon X-ray exposure, suggesting a mechanistic continuum between PDT and RDT in terms of molecular excitation and oxidative cytotoxicity [6]. Despite the clinical establishment of talaporfin as a photosensitizer, its radiosensitizing potential has not been systematically evaluated. Thus, exploring whether talaporfin can mediate RDT effects could provide an important translational bridge between PDT and RT.

Among photosensitizers, 5-ALA—induced protoporphyrin IX (PpIX) has been the most extensively studied in RDT contexts. PpIX preferentially accumulates in tumor cells and emits fluorescence upon photoexcitation, enabling intraoperative tumor visualization in PDD [21,22]. Because PpIX predominantly generates singlet oxygen, it also serves as an effective photosensitizer for PDT [23]. In contrast, RDT generates a broader ROS profile, including ^•^OH, reflecting photochemical pathways that differ from those seen when talaporfin is activated by visible light. Although talaporfin PDT is generally dominated by Type II singlet-oxygen formation, Type I processes can also occur, and X-ray irradiation appears to shift this balance toward radical species [8,24]. To our knowledge, this is the first study to assess talaporfin as a potential radiosensitizer and to evaluate the feasibility of talaporfin-mediated RDT. When comparing the photochemical properties of PpIX and talaporfin, the fluorescence quantum yield of PpIX (≈0.08) is higher than that of talaporfin (<0.001), whereas their singlet oxygen quantum yields are comparable (≈0.5–0.8) [12,13,25,26,27]. Both compounds show tumor selectivity, and intracellular accumulation can reach tens to hundreds of micromolar concentrations under comparable conditions [28,29]. However, their physicochemical and subcellular distributions differ markedly: PpIX is poorly soluble and localizes mainly to mitochondria, while talaporfin is water-soluble and predominantly lysosomal [30,31]. These localization and solubility differences likely lead to distinct ROS-mediated signaling and cell death pathways under irradiation. Although detailed subcellular localization beyond lysosomes was not examined in this study, future analyses including endoplasmic reticulum-specific markers will be important for delineating additional organelle-level contributions to talaporfin-mediated RDT. Because the 150-keV X-rays used in this study fall well below the energy threshold required for Cherenkov radiation, the observed ROS formation is unlikely to arise from Cherenkov-mediated excitation of talaporfin. Instead, the results are most plausibly explained by indirect activation processes associated with X-ray exposure. In particular, low-energy X-rays are known to generate reactive species in aqueous environments, and interactions between these species and talaporfin can promote Type I pathways, thereby producing a broader ROS spectrum than that expected under visible-light PDT, where talaporfin predominantly yields singlet oxygen through Type II reactions. Related radiation-assisted activation mechanisms—including Cherenkov-based pathways at higher photon or particle energies and non-optical excitation routes under keV X-ray irradiation—have been summarized in recent analyses of X-ray-driven photochemical activation [32].

Consistent with previous PDT studies reporting effective talaporfin activity at 10–30 µg/mL across multiple carcinoma and sarcoma models [33,34], we adopted a similar concentration range in our experiments. Talaporfin-based RDT induced significant cytotoxicity in both MIA PaCa-2 pancreatic cancer and U-251 MG glioma cells—two radioresistant lines of distinct origin [35,36,37]—indicating that its radiosensitizing activity is not cell-type specific. This enhanced cytotoxicity supports the hypothesis that RDT amplifies radiation-induced oxidative stress and may be particularly effective against tumors refractory to conventional RT. Although talaporfin-mediated RDT enhanced cytotoxicity across both cell lines, its radiosensitizing effect appeared less pronounced at higher X-ray doses. This is because at high radiation levels, direct radiation-induced DNA damage becomes the dominant lethal pathway, leaving little remaining capacity for additional enhancement by RDT. In contrast, radiosensitization is most evident in the low-to-intermediate dose range, where radiation lethality has not yet saturated and talaporfin-driven ROS generation can exert a measurable impact on clonogenic survival. PDT using talaporfin has been investigated in various tumor models in vivo, including TE-11R esophageal squamous-cell carcinoma, HCT-116 colorectal carcinoma, and C6 glioma. In these studies, talaporfin was typically administered at doses ranging from 2.5 to 10 mg/kg, followed by light irradiation 2 h after injection [34,38,39]. In our study, we administered 10 mg/kg of talaporfin and performed X-ray irradiation 2 h post-injection. The radiation regimen followed a fractionated protocol commonly employed in RDT, consisting of 3 Gy per day for three consecutive days (total dose = 9 Gy). Talaporfin was administered 2 h before each irradiation session, in reference to the established PDT protocol. Tumor regression was observed exclusively in the talaporfin-mediated RDT group, without apparent systemic toxicity. This selective tumor suppression with the combined treatment supports the interpretation that talaporfin functions as a radiosensitizer under X-ray exposure rather than as a direct cytotoxic agent.

To elucidate the molecular mechanisms underlying tumor regression, RNA-seq was performed on xenograft tumors collected 25 days after completion of combined TS + XT therapy. Because xenograft RNA contains both human (tumor) and mouse (host) transcripts, the dataset was computationally deconvoluted and analyzed separately to distinguish tumor-intrinsic and host-derived responses. A notable finding was the markedly high proportion of mouse reads in the most regressed sample, which reduced statistical power; therefore, genes with nominal significance (*p* < 0.01) were analyzed as supportive evidence. It should be noted that this analytical framework is based on a human tumor xenograft model established in immunosuppressed mice, which represents a so-called “two-species” system and does not fully recapitulate the clinical tumor-immune microenvironment. This model was intentionally adopted to enable evaluation of the direct tumor-intrinsic and radiosensitizing effects of talaporfin on human cancer cells in vivo, independent of host immune responses. Nevertheless, we acknowledge this as a limitation of the present study. Future investigations using syngeneic murine tumor models will be important to clarify immune-mediated and microenvironment-dependent effects of talaporfin-mediated RDT under more clinically relevant conditions.

In human tumors, transcriptional patterns reflected a shift from proliferative to stress-adaptive states. Upregulation of proteostasis and repair factors (*PSMD13*, *AKR1A1*, *PRIMPOL*) together with immune mediators (*CXCL1*, *MICA*) indicated sustained stress and immune activation, while suppression of mitochondrial and developmental regulators (*DMGDH*, *TBR1*) suggested metabolic downshift and loss of proliferative potential. Similar oxidative and proteostatic stress-associated transcriptional programs have been reported in porphyrin-based PDT models, in which ROS-mediated cellular stress responses constitute the core of treatment-induced gene networks [40]. Furthermore, large-scale integrative analyses of RT response across multiple cancer types have identified replication stress, DNA repair, and immune activation as central determinants of radiosensitivity [41], supporting the mechanistic relevance of these pathways in talaporfin-mediated RDT. In the mouse transcriptome, activation of stress-response genes (*Gstp1*, *Ddit3*) and metabolic regulators (*Rbks*, *Enho*) implied host adaptation and tissue recovery. Simultaneous repression of angiogenic and repair pathways (*Spon2*, *Msh6*, *Ndufs1*) pointed to reduced vascular and replicative activity within the regressing microenvironment. The elevated fraction of mouse RNA is unlikely to arise solely from technical contamination; rather, it likely reflects biological replacement of tumor tissue by stromal, immune, and endothelial cells during regression. This interpretation is supported by induction of host stress- and remodeling-related genes (*Ddit3*, *Gstp1*, *Spon2*), representing a molecular signature of tissue repair and immune infiltration consistent with the observed tumor regression. The data indicate that radiosensitization not only induced acute DNA and oxidative stress but also triggered durable cellular and microenvironmental remodeling that favors tumor resolution. Overall, these transcriptional patterns are consistent with a post-treatment state characterized by tumor regression, metabolic quiescence, and immune-mediated clearance rather than ongoing proliferation. Because RNA-seq was performed 25 days after irradiation, the transcriptomic changes observed here are more likely to reflect late-phase remodeling during tumor regression rather than acute injury, including vascular effects.

This study has several limitations. The use of immunodeficient nude mice restricts evaluation of immune-mediated mechanisms that may contribute to the antitumor effects of RDT. The sample size was relatively small, which may limit the statistical power for detecting subtle treatment effects. Furthermore, the study was not blinded during tumor measurement, potentially introducing observer bias, although standardized procedures were employed. Future studies using immunocompetent or orthotopic tumor models will be necessary to further validate the therapeutic potential and immune involvement of talaporfin-mediated RDT.

Taken together, our results provide a comprehensive framework for understanding the antitumor effects of talaporfin-mediated RDT. The treatment triggers X-ray-dependent ROS production, causing oxidative stress-induced apoptosis through mitochondrial and lysosomal damage, while simultaneously reprogramming the tumor microenvironment toward an immune-activated, reparative state. These findings highlight the unique potential of talaporfin as a radiosensitizer that combines direct cytotoxic effects with microenvironmental modulation. Given that talaporfin is already approved for PDT in lung and brain cancers, its translational pathway toward RDT could be relatively straightforward, including deep-seated or intrinsically radioresistant malignancies such as pancreatic cancer and glioma.

## 5. Conclusions

This study demonstrates that talaporfin can act as an effective radiosensitizer when activated by X-ray irradiation, establishing a mechanistic link between PDT and RDT. Talaporfin-mediated RDT elicited potent antitumor effects through ROS-dependent oxidative stress, leading to apoptosis and transcriptional reprogramming in both tumor and host compartments. RNA-seq analyses revealed coordinated activation of proteostatic and repair pathways in tumor cells, accompanied by stress and remodeling responses in host tissues, indicative of immune engagement and tissue repair during tumor regression. These findings suggest that talaporfin-mediated RDT induces not only acute cytotoxicity but also durable microenvironmental remodeling that favors tumor resolution. Given its clinical approval and safety profile as a photosensitizer, talaporfin represents a promising candidate for translation into RDT applications targeting deep-seated solid tumors.

## 6. Patents

The authors declare that details of intellectual property related to this study cannot be disclosed at this time.

## Figures and Tables

**Table 1:** Summary of tumor weights and proportions of species-derived RNA-seq reads in xenograft samples.

Sample	Tumor Weight (mg)	Human Reads	Mouse Reads	Mouse RNA Admixture (%)
NT-1	417.9	14,649,186	7,497,437	33.9
NT-2	199.5	15,478,986	7,992,317	34.1
NT-3	382.4	15,046,807	6,635,831	30.6
TS-1	410.0	17,728,354	7,719,592	30.3
TS-2	323.0	15,651,081	6,905,185	30.6
TS-3	187.0	19,227,210	6,786,295	26.1
XT-1	87.1	13,986,300	9,379,099	40.1
XT-2	121.6	17,221,493	8,601,562	33.3
XT-3	160.2	16,711,338	6,610,512	28.3
TS + XT-1	29.1	12,929,650	10,336,294	44.4
TS + XT-2	44.0	15,627,560	6,784,275	30.3
TS + XT-3	9.8	4,981,933	17,188,433	77.5

The proportion of mouse-derived reads (mouse read fraction) was used to estimate mouse RNA admixture, reflecting the degree of host tissue contamination. Notably, one TS + XT sample (TS + XT-3) exhibited a high mouse read fraction (77.5%), consistent with extensive tumor regression and increased host tissue content.

**Table 2:** Functional categorization of human and mouse genes differentially expressed between NT and TS + XT groups.

Group	Functional Category	Representative Genes	Interpretation
Human up regulated	Proteostasis/detoxification	*PSMD13*, *AKR1A1*, *PSMC4*	Protein quality control and oxidative stress defense
Human up regulated	Replication stress/DNA repair	*PRIMPOL*, *H2BC17*	Recovery from replication and DNA damage
Human up regulated	Inflammatory/immune response	*CXCL1*, *CXCL2*, *MICA*, *TMEM106A*	Cytokine-mediated inflammatory and immune activation
Human down regulated	Mitochondrial/redox & cofactor metabolism	*DMGDH*, *ATP23*, *COQ3*, *POR*, *NUBPL*	Oxidative/ETC activity curtailed
Human down regulated	Transcriptional/developmental programs	*TBR1*, *HOXD12*, *MORF4*, *ZNF624*, *TFCP2L1*, *EN1*, *SATB1*, *ZNF471*,	Stemness/development repressed
Human down regulated	Cilia/cytoskeleton & motility apparatus	*CCDC38*, *TMEM132B*, *MTCL1*, *DNAH1*, *CCDC159*	Diminished ciliogenesis/microtubule dynamics
Mouse up regulated	Integrated stress response (ISR)	*Gstp1*, *Slc39a3*, *Ddit3*, *Mief2*	ER/oxidative stress
Mouse up regulated	Metabolic remodeling	*Rbks*, *Yif1b*, *Enho*	Endocrine-metabolic reprogramming
Mouse down regulated	Angiogenesis/ECM remodeling	*Spon2*, *Adam11*, *Sema3a*, *Ank1*, *Myh10*	Vascular and stromal downregulation
Mouse down regulated	DNA repair/chromatin organization	*Setd1a*, *Msh6*, *Terf1*, *Kat5*	Reduced repair activity
Mouse down regulated	Mitochondrial/metabolic energy	*Cherp*, *Ndufs1*, *Vma21*	Lower respiratory metabolism

Genes showing nominal *p*  <  0.01 differences between NT and TS + XT were grouped according to biological function for both human (tumor) and mouse (host) compartments. Functional categories were manually curated based on Gene Ontology annotations. Representative genes and their putative biological interpretations are summarized.

## Data Availability

RNA-seq data have been deposited in the Gene Expression Omnibus (GEO) under the accession number GSE312155. All other data supporting the findings of this study are available from the corresponding author upon reasonable request.

## Supplementary Materials

The following supporting information can be downloaded at: https://www.mdpi.com/article/10.3390/biom15121748/s1, Figure S1: Correlation heatmaps of RNA-seq gene expression profiles in human and mouse compartments; Table S1: List of human genes upregulated between NT and TS + XT groups, Table S2: List of human genes downregulated between NT and TS + XT groups, Table S3: List of mouse genes upregulated between NT and TS + XT groups, Table S4: List of mouse genes downregulated between NT and TS + XT groups.

## Abbreviations

The following abbreviations are used in this manuscript:

5-ALA	5-Aminolevulinic acid
APF	3′-(p-Aminophenyl) fluorescein
DHE	Dihydroethidium
NPe6	Mono-L-aspartyl chlorin e6
PDD	Photodynamic diagnosis
PDT	Photodynamic therapy
PpIX	Protoporphyrin IX
ROS	Reactive oxygen species
RDT	Radiodynamic therapy
RT	Radiotherapy
