# Supplementary material for: Talaporfin Sodium as a Clinically Translatable Radiosensitizer in Radiodynamic Therapy
Source: Biomolecules. 2025 Dec 18;15(12):1748. doi: 10.3390/biom15121748 (PMC12730860; doi:10.3390/biom15121748)
Supplement: Supplementary file 1 [file biomolecules-15-01748-s001.zip › Supplementary Figure S1.pdf]

SUPPLEMENTARY FIGURE

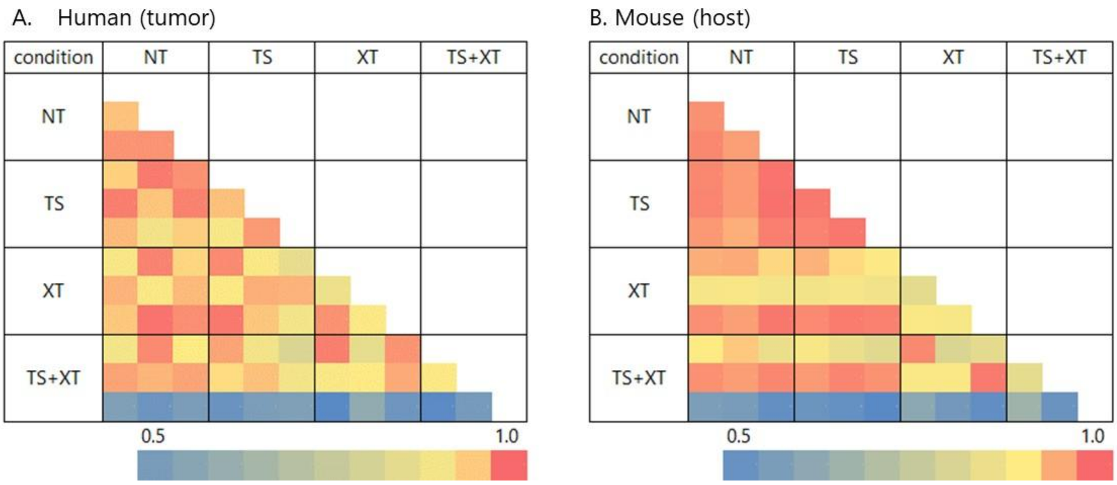

Supplementary Figure S1. Correlation heatmaps of RNA-seq gene expression profiles in human and mouse compartments. Transcriptomic correlations were calculated among samples within and across treatment conditions for the human (left, tumor compartment) and mouse (right, host compartment) datasets. Warmer colors indicate higher Pearson correlation coefficients, while cooler colors represent lower correlations. Strong intra-group correlations confirm the reproducibility of the RNA-seq data, whereas inter-group correlations progressively decreased from NT and TS to XT and TS+XT, indicating distinct transcriptional reprogramming induced by radiation and the radiosensitizer. One TS+XT sample showed relatively low correlation with other replicates, consistent with high mouse RNA admixture resulting from extensive tumor regression.
