# Supplementary material for: Talaporfin Sodium as a Clinically Translatable Radiosensitizer in Radiodynamic Therapy
Source: Biomolecules. 2025 Dec 18;15(12):1748. doi: 10.3390/biom15121748 (PMC12730860; doi:10.3390/biom15121748)
Supplement: Supplementary file 1 [file biomolecules-15-01748-s001.zip › Supplementary tables.pdf]

## SUPPLEMENTARY TABLES

**Supplementary Table S1.** List of human genes upregulated between NT and TS + XT groups

| Gene ID | Ratio vs NT | | | P value vs NT | | | Gene name |
|---|---|---|---|---|---|---|---|
| | 13 | XT | TS-XT | TS | XT | TS-XT | |
| ENSG00000185627 | 0.98 | 1.24 | 1.16 | 0.227 | 0.234 | 0.003 | PSMD13 |
| ENSG00000117448 | 1.01 | 1.02 | 1.24 | 0.904 | 0.837 | 0.005 | AKR1A1 |
| ENSG00000281221 | 1.02 | 1.39 | 1.25 | 0.791 | 0.219 | 0.008 | PSMC4 |
| ENSG00000163739 | 1.95 | 1.61 | 2.69 | 0.281 | 0.385 | 0.009 | CXCL1 |
| ENSG00000081041 | 1.04 | 1.24 | 1.97 | 0.805 | 0.672 | 0.003 | CXCL2 |
| ENSG00000236826 | 2.28 | 1.84 | 2.17 | 0.024 | 0.357 | 0.000 | LSM2 |
| ENSG00000164306 | 1.11 | 1.46 | 1.24 | 0.024 | 0.013 | 0.009 | PRIMPOL |
| ENSG00000274641 | 1.92 | 4.25 | 4.51 | 0.498 | 0.160 | 0.001 | H2BC17 |
| ENSG00000179111 | 0.88 | 2.70 | 4.33 | 0.660 | 0.078 | 0.005 | HES7 |
| ENSG00000167968 | 1.16 | 1.75 | 1.68 | 0.383 | 0.144 | 0.006 | DNASE1L2 |
| ENSG00000104826 | 1.60 | 5.25 | 7.26 | 0.372 | 0.216 | 0.000 | LHB |
| ENSG00000104140 | 0.97 | 1.39 | 1.76 | 0.947 | 0.438 | 0.008 | RHOV |
| ENSG00000233051 | 2.40 | 1.14 | 1.95 | 0.118 | 0.538 | 0.001 | MICA |
| ENSG00000184988 | 0.11 | 0.36 | 5.43 | 0.171 | 0.311 | 0.003 | TMEM106A |

**Supplementary Table S2.** List of human genes downregulated between NT and TS + XT groups

| Gene ID | Ratio vs NT | | | P value vs NT | | | Gene name |
|---|---|---|---|---|---|---|---|
| | 13 | XT | TS-XT | TS | XT | TS-XT | |
| ENSG00000132837 | 0.90 | 0.31 | 0.00 | 0.806 | 0.050 | 0.003 | DMGDH |
| ENSG00000161681 | 0.61 | 1.60 | 0.00 | 0.297 | 0.624 | 0.000 | SHANK1 |
| ENSG00000165972 | 0.35 | 0.46 | 0.00 | 0.186 | 0.348 | 0.009 | CCDC38 |
| ENSG00000166896 | 0.27 | 1.47 | 0.00 | 0.016 | 0.638 | 0.005 | ATP23 |
| ENSG00000172320 | 0.00 | 0.56 | 0.00 | 0.000 | 0.476 | 0.000 | OR5A1 |
| ENSG00000175189 | 0.55 | 0.46 | 0.00 | 0.056 | 0.024 | 0.001 | INHBC |
| ENSG00000178458 | 0.00 | 0.24 | 0.00 | 0.000 | 0.040 | 0.000 | H3P16 |
| ENSG00000197632 | 0.00 | 0.93 | 0.00 | 0.002 | 0.945 | 0.002 | SERPINB2 |
| ENSG00000198155 | 0.54 | 1.31 | 0.00 | 0.086 | 0.464 | 0.006 | ZNF876P |
| ENSG00000204305 | 0.43 | 0.08 | 0.00 | 0.102 | 0.007 | 0.003 | AGER |
| ENSG00000206305 | 0.00 | 0.00 | 0.00 | 0.005 | 0.005 | 0.005 | HLA-DQA1 |
| ENSG00000231292 | 0.68 | 0.19 | 0.00 | 0.405 | 0.013 | 0.000 | IGKV1OR2-108 |
| ENSG00000237522 | 0.90 | 1.17 | 0.00 | 0.737 | 0.895 | 0.007 | NONOP2 |
| ENSG00000244731 | 0.68 | 0.00 | 0.00 | 0.673 | 0.006 | 0.006 | C4A |
| ENSG00000248830 | 1.03 | 0.17 | 0.00 | 0.974 | 0.038 | 0.009 | ZNF807P |
| ENSG00000250995 | 2.07 | 1.61 | 0.00 | 0.393 | 0.225 | 0.000 | TMEM30BP1 |

| | | | | | | | |
|---|---|---|---|---|---|---|---|
| ENSG00000278616 | 1.24 | 1.04 | 0.00 | 0.428 | 0.956 | 0.007 | BEND3P3 |
| ENSG00000283703 | 0.00 | 0.00 | 0.00 | 0.009 | 0.009 | 0.009 | VSIG10L2 |
| ENSG00000291692 | 0.82 | 0.74 | 0.00 | 0.625 | 0.312 | 0.003 | PCDHB13 |
| ENSG00000186952 | 0.56 | 1.00 | 0.02 | 0.268 | 0.995 | 0.007 | TMEM232 |
| ENSG00000136535 | 0.29 | 1.15 | 0.07 | 0.015 | 0.816 | 0.000 | TBR1 |
| ENSG00000170178 | 0.51 | 0.32 | 0.09 | 0.092 | 0.035 | 0.010 | HOXD12 |
| ENSG00000139364 | 2.34 | 0.57 | 0.12 | 0.012 | 0.211 | 0.004 | TMEM132B |
| ENSG00000012124 | 1.51 | 0.76 | 0.13 | 0.655 | 0.572 | 0.003 | CD22 |
| ENSG00000162510 | 2.16 | 2.76 | 0.13 | 0.225 | 0.310 | 0.001 | MATN1 |
| ENSG00000234801 | 0.74 | 0.53 | 0.13 | 0.216 | 0.237 | 0.003 | MORF4 |
| ENSG00000159450 | 1.21 | 0.54 | 0.13 | 0.422 | 0.085 | 0.004 | TCHH |
| ENSG00000224999 | 0.99 | 0.53 | 0.14 | 0.985 | 0.214 | 0.001 | VTA1P1 |
| ENSG00000254221 | 0.99 | 0.59 | 0.15 | 0.984 | 0.153 | 0.001 | PCDHGB1 |
| ENSG00000038945 | 0.29 | 0.33 | 0.15 | 0.007 | 0.026 | 0.003 | MSR1 |
| ENSG00000120278 | 0.22 | 0.61 | 0.15 | 0.007 | 0.108 | 0.002 | PLEKHG1 |
| ENSG00000163501 | 1.46 | 0.14 | 0.16 | 0.351 | 0.008 | 0.009 | IHH |
| ENSG00000067601 | 1.34 | 0.87 | 0.18 | 0.233 | 0.399 | 0.004 | PMS2P4 |
| ENSG00000251595 | 0.45 | 0.37 | 0.19 | 0.133 | 0.002 | 0.004 | ABCA11P |
| ENSG00000215093 | 0.35 | 0.51 | 0.19 | 0.009 | 0.162 | 0.005 | EEF1A1P29 |
| ENSG00000278016 | 1.24 | 0.71 | 0.23 | 0.117 | 0.479 | 0.007 | TIGD5 |
| ENSG00000241549 | 0.72 | 0.40 | 0.23 | 0.621 | 0.122 | 0.003 | GUSBP2 |
| ENSG00000163599 | 0.69 | 0.93 | 0.23 | 0.091 | 0.735 | 0.004 | CTLA4 |
| ENSG00000275932 | 0.87 | 0.90 | 0.23 | 0.639 | 0.503 | 0.007 | DUSP14 |
| ENSG00000116981 | 0.79 | 0.63 | 0.24 | 0.389 | 0.098 | 0.006 | NT5C1A |
| ENSG00000197566 | 0.79 | 0.71 | 0.24 | 0.531 | 0.258 | 0.009 | ZNF624 |
| ENSG00000136943 | 1.10 | 0.51 | 0.27 | 0.402 | 0.035 | 0.005 | CTSV |
| ENSG00000116299 | 1.22 | 0.89 | 0.28 | 0.442 | 0.717 | 0.005 | ELAPOR1 |
| ENSG00000115112 | 1.14 | 0.50 | 0.28 | 0.546 | 0.072 | 0.007 | TFCP2L1 |
| ENSG00000184923 | 0.80 | 0.73 | 0.29 | 0.179 | 0.181 | 0.008 | NUTM2A |
| ENSG00000168502 | 0.98 | 0.64 | 0.31 | 0.888 | 0.254 | 0.002 | MTCL1 |
| ENSG00000183840 | 1.34 | 0.53 | 0.33 | 0.339 | 0.105 | 0.009 | GPR39 |
| ENSG00000029534 | 1.15 | 0.53 | 0.33 | 0.450 | 0.065 | 0.008 | ANK1 |
| ENSG00000170500 | 1.30 | 0.59 | 0.34 | 0.135 | 0.329 | 0.006 | LONRF2 |
| ENSG00000171812 | 0.82 | 0.37 | 0.35 | 0.094 | 0.004 | 0.003 | COL8A2 |
| ENSG00000151553 | 0.77 | 0.99 | 0.35 | 0.179 | 0.984 | 0.010 | FHIP2A |
| ENSG00000114841 | 0.88 | 0.94 | 0.37 | 0.482 | 0.716 | 0.010 | DNAH1 |
| ENSG00000274180 | 1.32 | 0.60 | 0.37 | 0.032 | 0.085 | 0.010 | NATD1 |
| ENSG00000213799 | 1.03 | 0.91 | 0.42 | 0.823 | 0.720 | 0.004 | ZNF845 |
| ENSG00000188051 | 1.19 | 0.79 | 0.43 | 0.475 | 0.405 | 0.003 | TMEM221 |

| | | | | | | | |
|---|---|---|---|---|---|---|---|
| ENSG00000072954 | 1.18 | 0.56 | 0.43 | 0.048 | 0.024 | 0.008 | TMEM38A |
| ENSG00000141469 | 0.41 | 0.68 | 0.44 | 0.002 | 0.141 | 0.003 | SLC14A1 |
| ENSG00000100344 | 1.46 | 0.93 | 0.46 | 0.460 | 0.790 | 0.005 | PNPLA3 |
| ENSG00000163064 | 1.10 | 0.84 | 0.50 | 0.712 | 0.415 | 0.010 | EN1 |
| ENSG00000182568 | 0.95 | 0.75 | 0.51 | 0.623 | 0.402 | 0.004 | SATB1 |
| ENSG00000219626 | 0.78 | 0.66 | 0.54 | 0.015 | 0.007 | 0.003 | FAM228B |
| ENSG00000183401 | 1.10 | 0.77 | 0.56 | 0.630 | 0.320 | 0.008 | CCDC159 |
| ENSG00000196263 | 1.30 | 1.14 | 0.57 | 0.405 | 0.848 | 0.004 | ZNF471 |
| ENSG00000105327 | 1.04 | 0.86 | 0.58 | 0.686 | 0.372 | 0.009 | BBC3 |
| ENSG00000111276 | 1.03 | 0.73 | 0.58 | 0.780 | 0.022 | 0.010 | CDKN1B |
| ENSG00000151413 | 0.80 | 0.73 | 0.59 | 0.069 | 0.033 | 0.010 | NUBPL |
| ENSG00000119720 | 1.18 | 0.99 | 0.71 | 0.213 | 0.775 | 0.008 | NRDE2 |
| ENSG00000172057 | 1.01 | 0.96 | 0.78 | 0.883 | 0.694 | 0.007 | ORMDL3 |
| ENSG00000132423 | 0.90 | 0.88 | 0.79 | 0.078 | 0.367 | 0.004 | COQ3 |
| ENSG00000127948 | 1.02 | 1.02 | 0.81 | 0.793 | 0.825 | 0.007 | POR |

**Supplementary Table S3.** List of mouse genes upregulated between NT and TS + XT groups

| Gene ID | Ratio vs NT | | | P value vs NT | | | Gene name |
|---|---|---|---|---|---|---|---|
| | 13 | XT | TS-XT | TS | XT | TS-XT | |
| ENSMUSG00000029136 | 1.06 | 0.96 | 1.22 | 0.687 | 0.772 | 0.003 | Rbks |
| ENSMUSG00000030588 | 1.13 | 1.30 | 1.25 | 0.012 | 0.090 | 0.009 | Yif1b |
| ENSMUSG00000060803 | 0.88 | 1.11 | 1.39 | 0.062 | 0.508 | 0.008 | Gstp1 |
| ENSMUSG00000046822 | 1.17 | 1.26 | 1.62 | 0.488 | 0.465 | 0.004 | Slc39a3 |
| ENSMUSG00000025408 | 0.89 | 1.55 | 1.84 | 0.524 | 0.264 | 0.010 | Ddit3 |
| ENSMUSG00000018599 | 0.61 | 1.44 | 2.20 | 0.121 | 0.236 | 0.008 | Mief2 |
| ENSMUSG00000028445 | 1.61 | 1.79 | 2.76 | 0.049 | 0.003 | 0.003 | Enho |
| ENSMUSG00000090812 | 1.41 | 0.82 | 4.68 | 0.226 | 0.718 | 0.010 | Samd15 |

**Supplementary Table S4.** List of mouse genes downregulated between NT and TS + XT groups

| Gene ID | Ratio vs NT | | | P value vs NT | | | Gene name |
|---|---|---|---|---|---|---|---|
| | 13 | XT | TS-XT | TS | XT | TS-XT | |
| ENSMUSG00000037747 | 0.10 | 0.17 | 0.00 | 0.002 | 0.002 | 0.000 | Phyhipl |
| ENSMUSG00000048699 | 0.00 | 0.59 | 0.00 | 0.000 | 0.257 | 0.000 | Krt90 |
| ENSMUSG00000068226 | 0.20 | 0.00 | 0.00 | 0.041 | 0.005 | 0.005 | Gm6723 |
| ENSMUSG00000071112 | 0.35 | 1.94 | 0.00 | 0.134 | 0.376 | 0.000 | Spx |
| ENSMUSG00000091421 | 1.48 | 0.69 | 0.00 | 0.212 | 0.514 | 0.008 | Gm4202 |
| ENSMUSG00000095614 | 0.15 | 0.46 | 0.00 | 0.005 | 0.138 | 0.000 | Gm6291 |
| ENSMUSG00000114738 | 0.77 | 0.83 | 0.00 | 0.660 | 0.773 | 0.009 | Gm41041 |
| ENSMUSG00000117507 | 0.00 | 0.53 | 0.00 | 0.000 | 0.225 | 0.000 | Gm50045 |

| | | | | | | | |
|---|---|---|---|---|---|---|---|
| ENSMUSG00000111272 | 0.77 | 0.40 | 0.07 | 0.430 | 0.062 | 0.001 | Gm7787 |
| ENSMUSG00000043460 | 0.94 | 0.72 | 0.08 | 0.868 | 0.566 | 0.008 | Elfn2 |
| ENSMUSG00000035775 | 0.83 | 0.58 | 0.08 | 0.786 | 0.367 | 0.003 | Krt20 |
| ENSMUSG00000006567 | 0.79 | 0.81 | 0.08 | 0.509 | 0.460 | 0.002 | Atp7b |
| ENSMUSG00000028701 | 0.96 | 0.92 | 0.10 | 0.863 | 0.855 | 0.002 | Lurap1 |
| ENSMUSG00000044254 | 0.57 | 1.09 | 0.10 | 0.086 | 0.880 | 0.007 | Pcsk9 |
| ENSMUSG00000111529 | 0.00 | 0.77 | 0.11 | 0.000 | 0.776 | 0.002 | Gm48141 |
| ENSMUSG00000115737 | 1.30 | 1.68 | 0.11 | 0.750 | 0.706 | 0.002 | Rpl19-ps3 |
| ENSMUSG00000033510 | 0.20 | 0.42 | 0.11 | 0.001 | 0.054 | 0.000 | Otud7a |
| ENSMUSG00000037379 | 1.03 | 0.54 | 0.13 | 0.960 | 0.153 | 0.004 | Spon2 |
| ENSMUSG00000020926 | 0.38 | 0.38 | 0.14 | 0.003 | 0.008 | 0.001 | Adam11 |
| ENSMUSG00000104577 | 0.51 | 0.60 | 0.14 | 0.054 | 0.122 | 0.003 | Gm6641 |
| ENSMUSG00000028528 | 0.47 | 0.47 | 0.15 | 0.053 | 0.073 | 0.002 | Dnajc6 |
| ENSMUSG00000112926 | 0.97 | 0.76 | 0.15 | 0.925 | 0.501 | 0.003 | Gm7172 |
| ENSMUSG00000049538 | 0.64 | 0.87 | 0.16 | 0.143 | 0.688 | 0.002 | Adamts16 |
| ENSMUSG00000020871 | 0.93 | 0.50 | 0.16 | 0.733 | 0.057 | 0.001 | Dlx4 |
| ENSMUSG00000029633 | 0.68 | 0.50 | 0.16 | 0.024 | 0.007 | 0.002 | Gm5578 |
| ENSMUSG00000106067 | 0.70 | 0.15 | 0.16 | 0.476 | 0.005 | 0.003 | Gm7902 |
| ENSMUSG00000008153 | 0.56 | 1.03 | 0.18 | 0.001 | 0.910 | 0.001 | Clstn3 |
| ENSMUSG00000042308 | 0.90 | 0.57 | 0.20 | 0.688 | 0.141 | 0.005 | Setd1a |
| ENSMUSG00000035566 | 0.47 | 0.62 | 0.20 | 0.031 | 0.064 | 0.001 | Pcdh17 |
| ENSMUSG00000040877 | 1.20 | 0.75 | 0.22 | 0.218 | 0.249 | 0.002 | Wdr25 |
| ENSMUSG00000028883 | 1.15 | 0.61 | 0.23 | 0.530 | 0.143 | 0.009 | Sema3a |
| ENSMUSG00000034235 | 1.09 | 0.61 | 0.26 | 0.478 | 0.174 | 0.009 | Usp54 |
| ENSMUSG00000111684 | 0.62 | 0.54 | 0.26 | 0.267 | 0.004 | 0.005 | Gm8543 |
| ENSMUSG00000031543 | 1.24 | 0.58 | 0.26 | 0.409 | 0.203 | 0.009 | Ank1 |
| ENSMUSG00000040213 | 0.89 | 1.00 | 0.28 | 0.591 | 0.988 | 0.009 | Kyat3 |
| ENSMUSG00000057457 | 0.83 | 0.58 | 0.28 | 0.294 | 0.092 | 0.010 | Phex |
| ENSMUSG00000075590 | 0.95 | 0.48 | 0.29 | 0.807 | 0.050 | 0.006 | Nrbp2 |
| ENSMUSG00000027883 | 1.19 | 0.55 | 0.30 | 0.103 | 0.063 | 0.007 | Gpsm2 |
| ENSMUSG00000005370 | 1.06 | 0.67 | 0.32 | 0.578 | 0.045 | 0.005 | Msh6 |
| ENSMUSG00000052488 | 0.74 | 0.57 | 0.33 | 0.230 | 0.145 | 0.008 | Cherp |
| ENSMUSG00000020900 | 1.21 | 0.91 | 0.36 | 0.511 | 0.632 | 0.004 | Myh10 |
| ENSMUSG00000025968 | 0.86 | 0.79 | 0.49 | 0.179 | 0.071 | 0.006 | Ndufs1 |
| ENSMUSG00000025925 | 0.57 | 0.87 | 0.52 | 0.027 | 0.731 | 0.002 | Terf1 |
| ENSMUSG00000085795 | 1.99 | 0.64 | 0.55 | 0.078 | 0.077 | 0.005 | Zfp703 |
| ENSMUSG00000003234 | 1.24 | 0.72 | 0.56 | 0.076 | 0.059 | 0.007 | Abcf3 |
| ENSMUSG00000024926 | 1.13 | 0.84 | 0.61 | 0.517 | 0.008 | 0.005 | Kat5 |
| ENSMUSG00000073131 | 0.65 | 0.86 | 0.79 | 0.040 | 0.343 | 0.006 | Vma21 |
